# A Case of Giant Squamous Cell Carcinoma of the Face Treated by Surgery Combined With Photodynamic Therapy

**DOI:** 10.1111/jocd.16791

**Published:** 2025-01-10

**Authors:** Fanfan She, Huiying Wang, Kongchao Yang, Xiaoming Qin, Ruzhi Zhang

**Affiliations:** ^1^ Department of Dermatology The Second Affiliated Hospital of Wannan Medical College Wuhu China; ^2^ Department of Dermatology The Third Affiliated Hospital of Soochow University Changzhou China

Dear Editor,

An 86‐year‐old woman presented with a rapidly growing right facial mass of more than 1 year duration, associated with bleeding, crusting, and occasional pain. Physical examination revealed an 8.0 cm × 8.0 cm mass on the right side of the face with clear margins, mild ulceration, and exudation (Figure 1A). Mild ectropion of the right eyelid was noted. Imaging studies showed no evidence of metastasis. Laboratory tests revealed that blood cell counts and coagulation function tests, including PT, APTT, INR, and D‐dimer, were within normal limits. In addition, LDH, an important prognostic marker for various tumors, was measured at 131 U/L, also within the normal range [1]. Based on the tumor size and the absence of distant metastases, the clinical stage was determined to be T3NxM0 [2]. Given the patient's age and tumor size, wide local excision (WLE) with a 6 mm margin was performed. However, due to the proximity of the tumor to the orbit, it was difficult to achieve a 6 mm margin in this area and the final margin was 2–4 mm (Figure 1B). Postoperative pathology revealed a moderately to poorly differentiated cutaneous squamous cell carcinoma (cSCC) with no evidence of perineural invasion (PNI) (Figure 1D,E). Immunohistochemistry showed positive staining for CK5/6, p40, Ki67, and p63 (Figure 2). Residual tumor cells were also found around the cutting edge. The wound surface was treated with three consecutive sessions of photodynamic therapy (PDT) starting on postoperative day 2, with each session separated by 1 week. It was treated locally with 20% 5‐aminolaevulinic acid (ALA) cream, followed by the application of a dark saran wrap for 3 h. Narrowband red light with a wavelength of 633 ± 10 nm was then applied at an intensity of 150 J/cm^2^. Each irradiation session lasted approximately 20 min, depending on patient tolerance. Secondary intentional healing (SIH) was chosen for reconstruction, with moist dressings used to facilitate wound healing. Dressings were changed every 2–3 days, starting with a layer of oil emulsion dressing mixed with antibiotic ointment, followed by clean gauze until wound healing was completed. By postoperative day 60, the majority of the wound had healed with no deformation of the surrounding tissue and minimal scarring (Figure 1C). A 6‐month follow‐up showed no recurrence.

**FIGURE 1:**
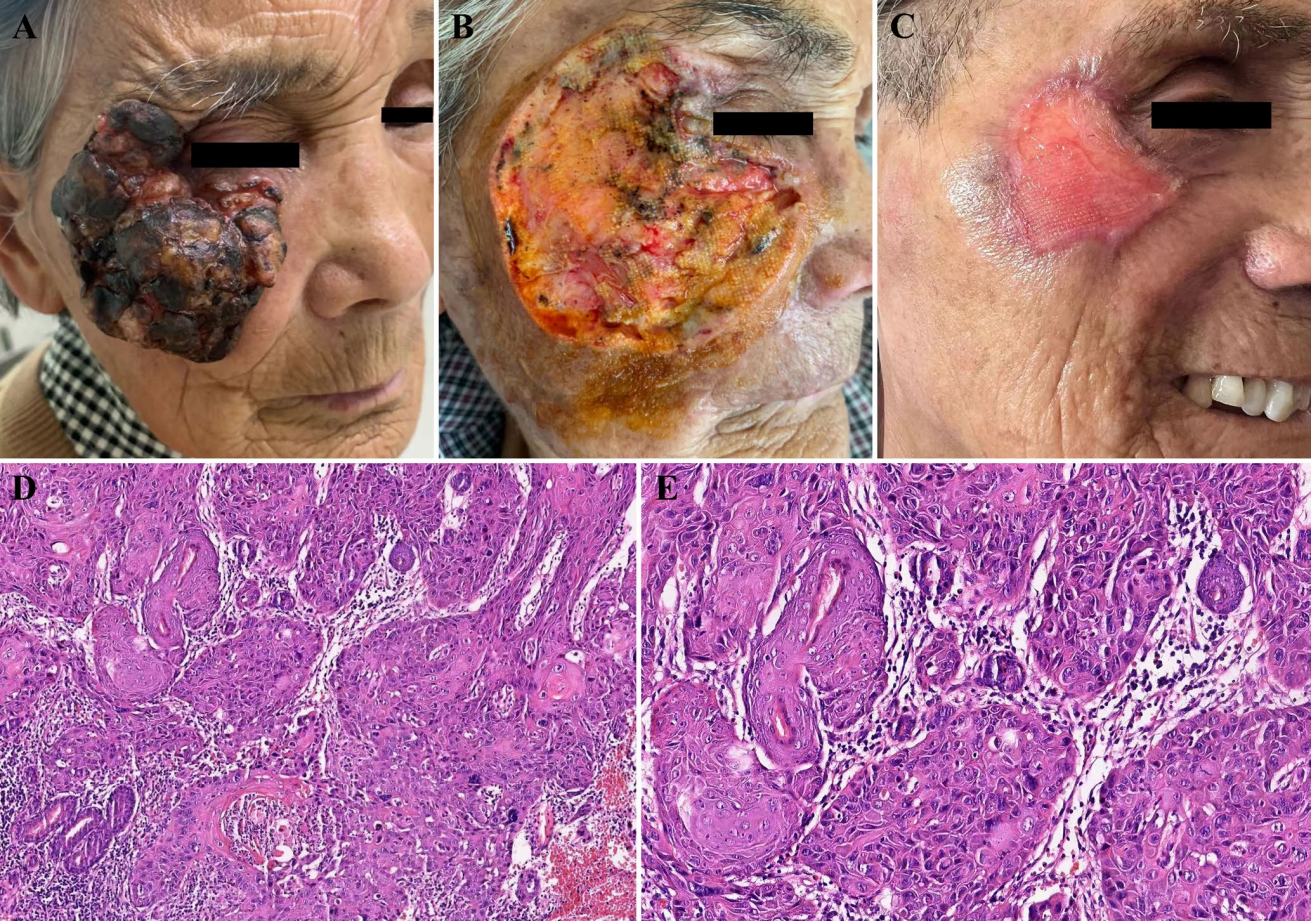
(A) Primary lesion. (B) After in situ resection. (C) On postoperative day 60, the wound was largely healed with no deformation of the surrounding tissue. (D) Histologic analysis shows moderately to poorly differentiated cSCC (H&E, original magnification ×100). (E) Higher magnification of histologic analysis (H&E, original magnification ×200).

**FIGURE 2:**
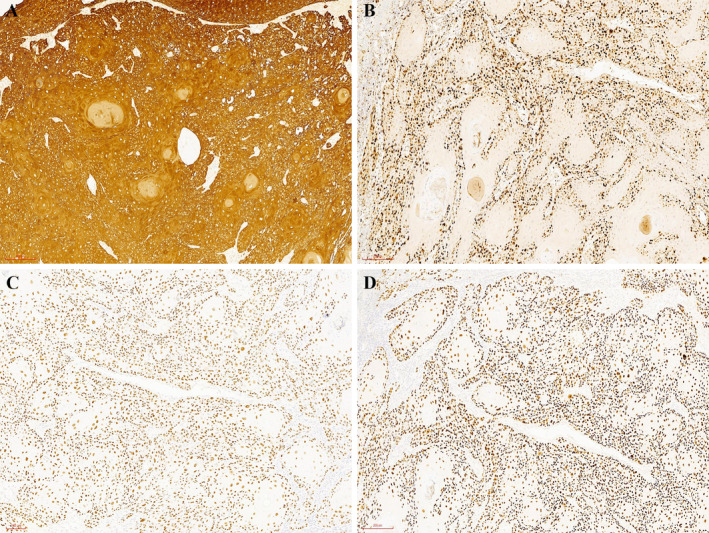
Immunohistochemistry shows positive staining for CK5/6 (A), Ki67 (B), p40 (C), and p63 (D) (100×).

Multivariate analysis of tumor characteristics identified five high‐risk factors as statistically independent prognostic indicators for cSCC: poor differentiation, PNI, tumor diameter > 2 cm, invasion of subcutaneous fat, and location in the ear, temple, or genital area. PNI, which is associated with disease‐specific mortality, can extend contiguously along with the perineural space to larger nerves before spreading proximally to the skull base [3, 4]. In this patient, the tumor was located near the temple area of the face and was more than 2 cm in diameter, presenting several high‐risk prognostic factors. However, she did not present with symptoms of nerve invasion such as facial numbness or paralysis. Given her age, general health, and tumor size, we opted for WLE rather than Mohs micrographic surgery to minimize operative time. However, due to the proximity of the tumor to the orbit, complete excision with a 6 mm margin was initially not feasible. Subsequently, after in situ tumor resection, we used three consecutive rounds of ALA‐PDT to reduce the residual tumor burden.

Photodynamic therapy has been reported to have excellent treatment effects on precancerous lesions or carcinoma in situ [5]. Although surgery remains the first‐line and most effective treatment for cSCC, the literature suggests that PDT may be used in special situations where surgery is not feasible, contraindicated, or not preferred by the patient after discussion of risks and benefits [6]. Previous reports have shown that ALA‐PDT after surgery effectively eradicated residual nasal SCC and resulted in favorable outcomes [7]. After careful consideration, we treated the giant cSCC with a combination of surgery and PDT. Contrary to the usual approach, we left the wound open and proceeded directly with PDT. This strategy enhances the binding of the photosensitizer to any residual tumor tissue, facilitating more complete tumor removal.

There are several reconstructive options for patients following the excision of cutaneous neoplasms of the head and neck, including primary closure, skin grafts, local, regional or free flaps, and SIH [8]. Given the size of the wound and the patient's reluctance to undergo skin flap grafting, we chose SIH for reconstruction. Healing by secondary intention is a viable option for appropriate wounds and offers several advantages: (1) saving time and costs associated with invasive procedures, (2) allowing better observation for signs of tumor recurrence, and (3) avoiding donor site scarring [8]. However, careful patient selection is essential when considering SIH. Studies have shown that wound contraction positively correlates not only with surface concavity but also with adjacent skin laxity, making aged skin often an ideal substrate for this approach. In addition, skin color and wound care are important factors to consider [8]. Patients must follow appropriate wound management practices to optimize esthetic outcomes. In our cases, moist wound healing was used to facilitate healing, with dressing changes every 2–3 days and vigilant monitoring for signs of exudation and tumor recurrence.

In conclusion, the combination of WLE with SIH and PDT has clear advantages in the treatment of skin tumors. This approach is worth considering, especially for special populations, although further patient experience is needed to confirm its feasibility and efficacy in treatment and reconstruction.

## Consent

Signed consent was obtained from the patient for the publication of the case details including publication of the images.

## Conflicts of Interest

The authors declare no conflicts of interest.

## Data Availability

The data that support the findings of this study are available from the corresponding author upon reasonable request.
